# Medical, nursing, and physician assistant student knowledge and attitudes toward climate change, pollution, and resource conservation in health care

**DOI:** 10.1186/s12909-020-02099-0

**Published:** 2020-06-23

**Authors:** Emma C. Ryan, Robert Dubrow, Jodi D. Sherman

**Affiliations:** 1 Environmental Health Sciences, Yale School of Public Health, New Haven, CT USA; 2 Environmental Health Sciences, Yale Center on Climate Change and Health, Yale School of Public Health, New Haven, CT USA; 3 Anesthesiology, Environmental Health Sciences, Yale School of Medicine, Yale School of Public Health, New Haven, CT USA

**Keywords:** Climate change, Pollution, Health care system, Medical student, Nursing student, Physician assistant student, Pollution prevention, Resource conservation

## Abstract

**Background:**

Climate change and pollution generated by the health care sector impose significant public health burdens. This study aimed to assess medical, nursing and physician assistant student knowledge and attitudes regarding climate change, pollution from the health care sector, and responsibility for resource conservation within professional practice.

**Methods:**

In February–March, 2018, medical, nursing, and physician assistant students at Yale University (1011 potential respondents) were sent a 17-question online Qualtrics survey. Data analysis included descriptive statistics, as well as Fisher’s exact test and logistic regression to assess associations between variables of interest and the personal characteristics of gender, age, geographic place of origin, school, and year in school (among medical students).

**Results:**

The response rate was 28% (280 respondents). 90% felt that physicians, nurses, and physician assistants have a responsibility to conserve resources and prevent pollution within their professional practice. 63% agreed or strongly agreed that the relationship between pollution, climate change, and health should be covered in the classroom and should be reinforced in the clinical setting. 57% preferred or strongly preferred reusable devices. 91% felt lack of time and production pressure, and 85% believed that lack of education on disease burden stemming from health care pollution, were barriers to taking responsibility for resource conservation and pollution prevention. Women and physician assistant students exhibited a greater commitment than men and medical students, respectively, to address pollution, climate change, and resource conservation in patient care and professional practice.

**Conclusion:**

We found that health professional students are engaged with the concept of environmental stewardship in clinical practice and would like to see pollution, climate change, and health covered in their curriculum. In order for this education to be most impactful, more research and industry transparency regarding the environmental footprint of health care materials and specific clinician resource consumption patterns will be required.

## Background

### Health care pollution as a public health problem

The Lancet Commission on Pollution and Health estimated that pollution-related diseases were responsible for 9 million premature deaths in 2015, accounting for 16% of all deaths worldwide [1]. Most of these deaths are linked to particulate air pollution, much of which is generated by the burning of fossil fuels, which also is the main source of greenhouse gas emissions causing climate change. Climate change has been named as the number one public health threat of the twenty-first century [2]. Furthermore, mitigating climate change has been identified as our greatest public health opportunity [3, 4], given that many mitigation strategies have significant health co-benefits, such as reductions in particulate air pollution [5].

Health professional leadership is essential to help reduce pollution, including mitigation of waste and environmental emissions from health care delivery itself. The US health care sector generates 10% of the nation’s greenhouse gas emissions and 9% of the nation’s criteria air pollutants [6]. Health care consumes large quantities of resources, and there is a concerning trend toward single-use disposable medical equipment and supplies without sufficient evidence of improved outcomes [7, 8]. The magnitude of disease burden stemming from health care waste and pollution is comparable to that of medical errors as first reported by the Institute of Medicine [9], suggesting that health care pollution prevention is an issue of comparable import to patient safety efforts [6].

This growing understanding of health sector pollution is causing some health systems, hospitals, and health professionals to recognize their role as environmental stewards, to work to reduce their waste and environmental emissions, and to serve as environmental health educators for patients [6, 10–12]. Macpherson and Hill argue that promoting sustainability is part of the ethical responsibility of physicians and health organizations to protect and promote health [13]. Given that 62% of Americans believe climate change to be a health issue [14], and given the trusted position of health professionals within society, health professionals have been identified as a key resource for education and advocacy work on the health effects of pollution and climate change [12, 15]. Groups such as the Medical Society Consortium on Climate and Health, whose organization members encompass more than 500,000 physicians, are working to increase physician engagement to both mitigate climate change and adapt to its health impacts [16].

### Health professional understanding of climate change, pollution, and health

Previous research has examined physician knowledge and attitudes about climate change and health, medical waste, and duty to educate patients [17–22]. Physicians have identified a need for improved health professional education to prepare them to respond to climate change related harm and to better communicate with patients, the public, and policy makers [17, 23, 24]. A recent study found that medical students in China understood the health impacts of climate change but did not feel adequately prepared to address these impacts [25]. In 2016, an unpublished study conducted by George Mason University examined medical students’ attitudes and knowledge regarding climate change and health as well as how much they valued incorporating climate change and health education into the curriculum at four medical schools [26]. The results of that work echoed calls to incorporate climate change and health education into medical school curricula to prepare future physicians to address health needs [27–29]. Core objectives for education about climate change and health and the environmental impact of the health care sector have been developed [23], but currently there is no health professional education mandate.

This project surveyed health professional students – medical, nursing, and physician assistant – at Yale University about their knowledge and attitudes regarding pollution from the health care sector itself, climate change, and responsibility for resource conservation within professional practice.

## Methods

We conducted an anonymous cross-sectional survey of health professional students at Yale University. At the time of the survey, climate change, pollution and resource conservation in the health care industry were not included in the curriculum. This population was chosen given the authors’ connections within the university and the feasibility of distributing the survey to the population. The survey was designed to assess knowledge, attitudes and opinions about climate change and health, pollution generated by the health care sector, and health professional responsibility. To inform development of the survey instrument, previous surveys of physicians and medical students regarding climate change and pollution were reviewed [17–21]. Many of the questions from these surveys (specifically questions 2, 4, 6, 9, 12, 13, 14, 15, 16) were adapted for use in the survey instrument. In addition, the authors developed questions (specifically questions 3, 5, 7, 8, 10, 11, 17) to address concepts for which questions were not present in the existing surveys (see the complete survey in the Supplemental Material). Dr. Mona Sarfaty and Dr. Edward Maibach, authors of several previous surveys [17–19, 22] provided input on wording of some questions. The final question (question 17 – see Supplemental Material) allowed students to provide open-ended feedback and comments.

The survey questions were pilot tested on members of the Yale Physicians for Social Responsibility Medical Student Interest Group, a small group of medical students already interested in climate change, whose oral feedback was used to edit the survey for length and clarity. The final online survey, constructed using Qualtrics, contained 17 questions, and required approximately 7 to 8 min to complete. Qualtrics was chosen given the platform’s capabilities regarding security, anonymity, and prevention of duplicate entries; ease of access through the university’s license; and ease of distribution.

The target population was all medical, nursing, and physician assistant students at Yale University (*N* = 1011, including 562 medical students, 372 nursing students, and 77 physician assistant students). An email message was sent to the target population with an online link to the survey a total of four times during February and March 2018. The principal investigator sent the emails to medical and physician assistant students, while the Yale School of Nursing Office of the Dean distributed the survey to nursing students through an all-student newsletter. Each respondent was only able to complete the survey once. Following completion of the survey, respondents had the option to provide their email address via an anonymous link to be entered into a lottery for one of five $100 Amazon gift cards.

Although the primary objective of the survey was descriptive, we also assessed potential associations between 30 variables of interest and the personal characteristics of gender, age, geographic place of origin, school, and year in school (among medical students) using Monte Carlo simulations to estimate Fisher’s exact test *p*-values, using SAS 9.4. To correct for multiple comparisons, associations were considered to be significant at the Bonferonni correction level of 0.002. We then conducted bivariate logistic regression analyses for significant relationships.

We also conducted bivariate logistic regression analyses for a priori relationships of interest: 1) knowledge of US greenhouse gas emissions emitted by the health care sector as predicted by a) concern about pollution stemming from the healthcare industry and b) concern about the health impacts of climate change; 2) belief that health professionals have a responsibility to conserve resources and prevent pollution within their professional practice as predicted by a) knowledge of the percentage of US greenhouse gas emissions emitted by the health care sector, b) knowledge of the burden of disease associated with pollution from the health care sector, and c) age, gender, geographic place of origin, school, and year in school (among medical students); and 3) belief that health professionals have an important role to play in educating patients and the public about the impacts of pollution and climate change on health as predicted by age, gender, geographic place of origin, school, and year in school (among medical students).

Based on findings of the bivariate logistic regression models, we conducted several multivariate logistic regression analyses.

This study was granted exemption status by the Yale University Institutional Review Board.

## Results

### Sample population

A total of 280 surveys (172 from medical students, 59 from nursing students, 49 from physician assistant students) were collected from 1011 potential respondents (28% response rate), with 257 respondents (25%) answering all survey questions. Answers from partially completed surveys (N = 23) were included in the analysis. Response rates for fully completed surveys varied considerably among health professions: 158/562 = 28% for medical students; 53/372 = 14% for nursing students; and 46/77 = 60% for physician assistant students.

The majority (70%) of respondents were between the ages of 25 and 34 years and were female (67%), although this varied by school (Table 1). Medical student respondents were 54% female compared with 48% of all medical students; nursing student respondents were 98% female compared with 83% of all nursing students; and physician assistant student respondents were 76% female compared with 73% of all physician assistant students [30]. Connecticut was the home state of 20% of respondents, with 73% of respondents from US states or territories other than Connecticut, and 7% of respondents from outside the US (Table 1).

**Table 1:** Selected demographic characteristics of respondents

Demographic Characteristics of Study Sample				
	Medical Students *(Question N = 158)*	Nursing Students *(Question N = 53)*	Physician Assistant Students *(Question N = 46)*	Combined Students *(Question N = 257)*
**Age**	% (N)	% (N)	% (N)	% (N)
*18–24*	29.7 (47)	16.9 (9)	23.9 (11)	26.1 (67)
*25–34*	69.6 (110)	75.5 (40)	63.0 (29)	69.7 (179)
*35–44*	0.6 (1)	3.8 (2)	10.9 (5)	3.1 (8)
*45 or above*	0.0 (0)	3.8 (2)	2.2 (1)	1.2 (3)
**Gender**				
*Male*	44.3 (70)	1.9 (1)	17.4 (8)	30.7 (79)
*Female*	54.4 (86)	98.1 (52)	76.1 (35)	67.3 (173)
*Other/Neither*	0.0 (0)	0.0 (0)	2.2 (1)	0.4 (1)
*Prefer not to answer*	1.3 (2)	0.0 (0)	4.3 (2)	1.6 (4)
**Home state or country**	Medical Students *(Question N = 156)*	Nursing Students *(Question N = 53)*	Physician Assistant Students *(Question N = 45)*	Combined Students *(Question N = 254)*
*Connecticut*	17.3 (27)	30.2 (16)	15.6 (7)	19.7 (50)
*US, outside Connecticut*	72.4 (113)	67.9 (36)	82.2 (37)	73.2 (186)
*Outside US*	10.3 (16)	1.9 (1)	2.2 (1)	7.1 (18)

### Survey responses

#### Concern regarding health impacts of pollution and climate change

The majority of respondents demonstrated concern for the health impacts of pollution and climate change: 94% either agreed or strongly agreed that they are concerned about the health impacts of climate change; 77% either agreed or strongly agreed that they are concerned about pollution from the health care industry; and 90% agreed or strongly agreed that health professionals have a responsibility to conserve resources and prevent pollution within their professional practice (Table 2).

**Table 2:** Summary of respondents’ knowledge and opinions regarding the health impacts of health sector pollution and climate change

Knowledge and opinions regarding health impacts of health sector pollution and climate change *Responses shown are % of respondents who “agree” or “strongly agree,” unless otherwise noted*				
	Medical Students % (N)	Nursing Students % (N)	Physician Assistant Students % (N)	Combined Students % (N)
I am concerned about the health impacts of climate change				
*Agree or strongly agree*	93.3 (153) *(Question N = 164)*	94.6 (52) *(Question N = 55)*	95.7 (44) *(Question N = 46)*	93.9 (191) *(Question N = 265)*
I am concerned about pollution stemming from the health care industry.				
*Agree or strongly agree*	70.7 (118) *(Question N = 167)*	82.1 (46) *(Question N = 56)*	93.5 (43) *(Question N = 46)*	76.9 (207) *(Question* *N = 269)*
Of all US greenhouse gas emissions, what percentage do you think is emitted by the health care sector?				
*% respondents correctly identifying “10%”*	33.5 (55) *(Question N = 164)*	47.3 (26) *(Question N = 55)*	43.5 (20) *(Question N = 46)*	38.1 (101) *(Question N = 265)*
The recently reported disease burden associated with health care sector pollution is of the same order of magnitude as the disease burden associated with medical errors first reported by the Institute of Medicine (To Err is Human).				
*Agree or strongly agree*	22.4 (37) *(Question N = 165)*	43.6 (24) *(Question N = 55)*	32.6 (15) *(Question N = 46)*	28.6 (76) *(Question N = 266)*
Physicians/Nurses/Physician Assistants and health professionals have a responsibility to conserve resources and prevent pollution within their professional practice.				
*Agree or strongly agree*	88.3 (143) *(Question N = 162)*	92.5 (49) *(Question N = 53)*	95.7 (44) *(Question N = 46)*	90.4 (236) *(Question N = 261)*

#### Knowledge of impact of health care sector emissions

Only 38% of respondents correctly identified that 10% of US greenhouse gas emissions are emitted by the health care sector, with 57% underestimating these emissions. Fewer than a third of respondents (29%) knew that the recently reported disease burden associated with health care sector pollution is of the same order of magnitude as the disease burden associated with medical errors (Table 2).

#### Including education on the relationship between pollution, climate change, and health in the curriculum

Two-thirds (67%) of respondents either agreed or strongly agreed that it is important to understand the relationship between pollution, climate change, and health so they can help their patients, while one-third (31%) of all respondents either agreed or strongly agreed that, although it is important to understand this issue, it is not pertinent to patient care (Table 3).

**Table 3:** Summary of respondents’ opinions on the inclusion of education on the relationship between pollution, climate change, and health in their curriculum

Opinions on inclusion of pollution, climate change, and health in the curriculum *Responses shown are % of respondents who*” *agree” or*” *strongly agree” with the each statement following the question “How much do you agree or disagree with each of the following statements about including education on the relationship between pollution, climate change, and health in the medical/nursing/physician assistant school curriculum?*				
	Medical Students *(Question N = 162)*	Nursing Students *(Question N = 53)*	Physician Assistant Students *(Question N = 46)*	Combined Students *(Question N = 261)*
	% (N)	% (N)	% (N)	% (N)
*It is important to understand this issue so I can help my patients*	60.5 (98)	81.1 (43)	73.9 (34)	67.1 (175)
*It is important to understand this issue, but it isn’t pertinent to patient care*	39.5 (64)	26.4 (14)	13.0 (6)	32.2 (84)
*I feel I can learn about this issue through observation in clinical practice, not classroom-based learning*	14.2 (23)	21.2 (11)	15.2 (7)	15.8 (41)
*I feel we should cover this issue in the classroom and it should be reinforced in the clinical setting*	57.4 (93)	71.7 (38)	71.7 (34)	62.8 (164)
*The training and testing we receive in medical school already requires a lot of my attention, there is no time to learn about this issue*	30.2 (49)	24.5 (13)	34.8 (16)	29.9 (78)
*I think there is a role for physicians/nurses/physician assistants in addressing this issue, but it is not my personal interest area*	57.4 (93)	39.6 (21)	41.3 (19)	50.9 (133)
*It is important to understand this issue because physicians/nurses/physician assistants have an important role to play in educating patients and the public about the impacts of pollution and climate change on health*	69.8 (113)	86.8 (46)	91.3 (42)	77.0 (201)
*Pollution and climate change should not be included in the medical/nursing/physician assistant school curriculum*	16.7 (27)	11.3 (6)	10.9 (5)	14.6 (38)

Almost two-thirds (63%) of respondents agreed or strongly agreed that the relationship between pollution, climate change, and health should be covered in the classroom and should be reinforced in the clinical setting, whereas 16% agreed or strongly agreed that they can learn about this issue through observation in clinical practice, not in classroom-based learning (Table 3). About one-third (30%) of respondents agreed or strongly agreed they do not have time to learn about this in school given that that the training and testing they receive already requires a lot of attention, and nearly one-sixth (15%) of respondents either agreed or strongly agreed that pollution and climate change should not be included in the curriculum (Table 3).

Approximately three-quarters (77%) of respondents either agreed or strongly agreed that it is important to understand the relationship between pollution, climate change, and health because health professionals have an important role to play in educating patients and the public about the impacts of pollution and climate change on health (Table 3). However, about one-half (51%) of respondents either agreed or strongly agreed that there is a role for health professionals in addressing this issue, but it is not their personal interest area (Table 3).

#### Preferences regarding reusable vs. disposable medical devices

More than half (57%) of respondents preferred or strongly preferred reusable devices, while one-fifth (20%) of respondents preferred or strongly preferred disposable devices, and about one-fifth (23%) of respondents had no preference (Table 4). About two-fifths (43%) of respondents agreed or strongly agreed with the incorrect statement that there is strong evidence that single-use disposable medical devices meaningfully reduce infection risk (Table 4).

**Table 4:** Summary of respondents’ preferences and knowledge regarding use of reusable and disposable medical devices

Preferences and knowledge on reusable and disposable medical devices				
	Medical Students % (N)	Nursing Students % (N)	Physician Assistant Students % (N)	Combined Students % (N)
**Medical device preference**				
*Prefer or strongly prefer reusable*	52.2 (84)	64.2 (34)	67.4 (31)	57.3 (149)
*No preference*	26.1 (42)	13.2 (7)	21.7 (10)	22.7 (59)
*Prefer or strongly prefer disposable*	21.7 (35)	22.6 (12)	10.9 (5)	20.0 (52)
	*(Question N = 161)*	*(Question N = 53)*	*(Question N = 46)*	*(Question N = 260)*
**Ranked #1 in preference determination**				
*Infection control*	53.4 (86)	52.8 (28)	54.4 (25)	53.5 (139)
*What’s available in my institution*	33.5 (54)	16.9 (9)	28.3 (13)	29.2 (76)
*Environmental sustainability*	4.9 (8)	11.3 (6)	10.9 (5)	7.3 (19)
*Habit*	4.4 (7)	15.1 (8)	2.2 (1)	6.2 (16)
*Concern for cost*	3.7 (6) *(Question N = 161)*	3.8 (2) *(Question N = 53)*	4.4 (2) *(Question N = 46)*	3.9 (10) *(Question N = 260)*
**There is strong evidence that use of single-use disposable medical devices meaningfully reduces infection risk.**				
*Agree or strongly agree*	43.1 (69) *(Question N = 160)*	50.9 (27) *(Question N = 53)*	34.8 (16) *(Question N = 46)*	43.2 (112) *(Question N = 259)*

Infection control was ranked as most important in determining reusable vs. disposable medical device preference by about half (53%) of respondents; what is available in the institution was ranked as most important by about one-third (29%) of respondents; environmental sustainability was ranked as most important by 7% of respondents; habit was ranked as most important by 6% of respondents; and concern for cost was ranked as most important by 4% of respondents (Table 4).

#### Ways to promote resource conservation and pollution prevention

Most respondents agreed or strongly agreed that the following are important ways to promote resource conservation and pollution prevention (Table 5): industry transparency about environmental footprint of supplies, procedures and services (89%); tracking medical device and supply utilization to guide resource conservation performance improvement (85%); evidence-based recommendations on minimizing unnecessary procedures and services (94%); and patient-centered conversations about end-of-life planning and advanced directives to reduce unwanted care (82%). When asked about ways to promote resource conservation and pollution prevention, only 13% of respondents said they agreed or strongly agreed that resource conservation and pollution prevention are not a health professional’s responsibility (Table 5).

**Table 5:** Summary of respondents’ opinions regarding ways to promote resource conservation and pollution prevention within their professional practice and barriers to doing so

Opinion on ways to address resource conservation and pollution prevention *Responses shown are % of respondents who*” *agree” or*” *strongly agree” with the statement*				
	Medical Students % (N)	Nursing Students % (N)	Physician Assistant Students % (N)	Combined Students % (N)
**Important ways for physicians/nurses/physician assistants to promote resource conservation and pollution prevention**				
*Industry transparency about environmental footprint of supplies, procedures and services*	88.8 (142)	86.8 (46)	89.1 (41)	88.4 (229)
*Track medical device and supply utilization, to guide resource conservation performance improvement*	87.5 (140)	79.3 (42)	82.6 (38)	84.9 (220)
*Evidence-based recommendations on minimizing unnecessary procedures and services (*e.g.*, as with the Choosing Wisely Campaign)*	95.0 (152)	92.5 (49)	93.5 (43)	94.2 (244)
*Patient-centered conversations about end-of-life planning and advanced directives to reduce unwanted care*	85.0 (136)	90.6 (48)	91.3 (42)	87.3 (226)
*Resource conservation and pollution prevention are not a physician’s responsibility*	10.0 (16) *(Question N = 160)*	16.9 (9) *(Question N = 53)*	17.4 (8)	12.7 (33) *(Question N = 259)*
**Important barriers that inhibit physicians/nurses/physician assistants from taking responsibility for resource conservation and pollution prevention**				
*Lack of education on disease burden stemming from health care pollution*	84.2 (133)	88.7 (47)	84.8 (39)	85.2 (219)
*Lack of time/production pressure leading to inefficient utilization of resources*	91.8 (145)	90.6 (48)	91.3 (42)	91.4 (253)
*Unrealistic expectations of infection risk reduction*	35.5 (56)	52.8 (28)	39.1 (18)	39.7 (102)
*Resource conservation and pollution prevention are not a physician’s/nurse’s/physician assistant’s responsibility*	20.3 (32) *(Question N = 158)*	18.9 (10) *(Question N = 53)*	8.7 (4) *(Question N = 46)*	17.9 (46) *(Question N = 257)*

#### Barriers to taking responsibility for resource conservation and pollution prevention

Most respondents agreed or strongly agreed that two important barriers inhibiting health professionals from taking responsibility for resource conservation and pollution prevention are a lack of education on disease burden stemming from health care pollution (85%) and lack of time and production pressure leading to inefficient utilization of resources (91%) (Table 5). Two-fifths (40%) of respondents also agreed or strongly agreed that unrealistic expectations of infection risk reduction are an important barrier to environmental stewardship (Table 5). When asked about barriers to taking responsibility for resource conservation and pollution prevention, 18% of respondents agreed or strongly agreed that resource conservation and pollution prevention are *not* a physician/nurse/physician assistant’s responsibility (Table 5).

### Significant relationships between personal characteristics and responses

We identified five associations that were significant according to the Bonferroni-corrected Fisher’s exact test. For each of these associations, we conducted logistic regression analyses (Table 6). Compared with students aged ≥25 years, students aged 18–24 years were 0.35 times as likely to disagree with the incorrect statement that “there is strong evidence that use of single-use disposable medical devices meaningfully reduces infection risk” (i.e., older students were more likely to answer the question correctly; *p* = 0.0003).

**Table 6:** Logistic regression models for relationships between personal characteristics and responses that were significant according to the Bonferroni-corrected Fisher’s exact test. Each model is bivariate, with one predictor variable and one outcome variable

Logistic regression models for significant associations according to the Bonferroni-corrected Fisher’s exact test			
**Outcome variable**	**Predictor variable**	**Odds ratio (95% CI)**	***p*-value**
**Disagreement**^a^**with statement:*There is strong evidence that use of single-use disposable medical devices meaningfully reduces infection risk.***	**Age** (*n* = 254)		0.0003
	18–24	0.35 (0.20, 0.63)	
	≥25	1.00 (ref)	
**Disagreement**^a^**with statement:*It is important to understand this issue [pollution, climate change, and health], but it isn’t pertinent to patient care.***	**Gender** (*n* = 252)		0.0004
	Female	2.72 (1.55, 4.75)	
	Male	1.00 (ref)	
**Disagreement**^a^**with statement*: It is important to understand this issue [pollution, climate change, and health], so I can help my patients.***	**Gender** (*n* = 252)		< 0.0001
	Female	0.31 (0.18, 0.55)	
	Male	1.00 (ref)	
	**Year in Med School** (*n* = 162)		0.093
	1	0.71 (0.24, 2.14)	
	2	1.57 (0.53, 4.65)	
	3	1.64 (0.50, 5.31)	
	4	2.46 (0.87, 6.97)	
	5 (research year)	1.00 (ref)	

^a^Disagreement is defined as: Strongly disagree, Disagree, or Neither agree nor disagree

Compared with men, women were 2.72 times as likely to disagree with the statement that “It is important to understand this issue [pollution, climate change, and health], but it isn’t pertinent to patient care” (i.e., women were more likely to believe that understanding of pollution, climate change, and health is pertinent to patient care; *p* = 0.0004). Compared with men, women were 0.31 times as likely to disagree with the statement that “it is important to understand this issue [pollution, climate change, and health] so I can help my patients” (i.e., women were more likely to agree with this statement; *p* < 0.0001). Although year in medical school was significantly associated with this statement according to the Fisher’s exact test, the association was not significant in the logistic regression model. Finally, professional school was associated with “it is important to understand this issue [pollution, climate change, and health] because physicians/nurses/physicians assistants have an important role to play in educating patients and the public about the impacts of pollution and climate change on health.” The logistic regression model for this association, which was one of our a priori relationships of interest, is presented in the next section.

### A priori relationships of interest

We conducted logistic regression analyses for a priori relationships of interest (Table 7) and found the following significant results (*p* < 0.05). Compared with students who strongly agreed that they were concerned about pollution stemming from the healthcare industry, students who disagreed or strongly disagreed that they were concerned about pollution stemming from the healthcare industry were 5.39 times as likely to not know the percentage of US greenhouse gas emissions emitted by the health care sector and students who neither agreed nor disagreed were 2.38 times as likely (*p* = 0.014).

**Table 7:** Logistic regression models for a priori relationships of interest. Each model is bivariate, with one predictor variable and one outcome variable

Logistic regression models for a priori relationships of interest			
**Outcome variable**	**Predictor variable**	**Odds ratio (95% CI)**	***p*-value**
**Incorrect answer to the question:*Of all US greenhouse gas emissions, what percentage do you think is emitted by the health care sector?***	**Concerned about pollution stemming from the health care industry** (*n* = 266)		0.014
	Disagree / Strongly disagree	5.39 (1.46, 19.84)	
	Neither agree nor disagree	2.38 (1.07, 5.29)	
	Agree	1.51 (0.85, 2.66)	
	Strongly Agree	1.00 (ref)	
	**Concerned about the health impacts of climate change.** (*n* = 266)		0.52
	Disagree	0.99 (0.16, 6.08)	
	Neither agree nor disagree	2.97 (0.62, 14.16)	
	Agree	1.09 (0.62, 1.93)	
	Strongly Agree	1.00 (ref)	
**Disagreement**^a^**with statement:*Physicians and health professionals have a responsibility to conserve resources and prevent pollution within their professional practice.***	**Knowledge of percentage of US greenhouse gas emissions emitted by the health care sector** (*n* = 261)		0.27
	Incorrect	1.64 (0.66, 4.09)	
	Correct	1.00 (ref)	
	**Knowledge of burden of disease associated with pollution from the health care sector** (*n* = 261)		0.12
	Incorrect	2.26 (0.75, 6.82)	
	Correct	1.00 (ref)	
**Disagreement**^a^**with statement:*It is important to understand this issue [pollution, climate change, and health] because physicians/nurses/physician assistants have an important role to play in educating patients and the public about the impacts of pollution and climate change on health.***	**Age (years)** (*n* = 254)		0.85
	18–24	0.94 (0.48, 1.83)	
	≥25	1.00 (ref)	
	**Gender** (*n* = 252)		0.0038
	Female	0.40 (0.22, 0.74)	
	Male	1.00 (ref)	
	**Geographic place of origin** (*n* = 254)		0.22
	Connecticut	1.22 (0.37, 4.02)	
	Other state	0.67 (0.22, 1.99)	
	Outside US	1.00 (ref)	
	**School** (*n* = 261)		0.0008
	Medical	4.55 (1.55, 13.39)	
	Nursing	1.60 (0.44, 5.85)	
	Physician Assistant	1.00 (ref)	
	**Year in Medical School** (*n* = 162)		0.15
	1	0.92 (0.28, 2.99)	
	2	1.19 (0.36, 3.93)	
	3	1.12 (0.30, 4.13)	
	4	2.74 (0.91, 8.25)	
	5 (research year)	1.00 (ref)	
**Disagreement**^a^**with statement:*Physicians and health professionals have a responsibility to conserve resources and prevent pollution within their professional practice.***	**Age (years)** (*n* = 254)		0.21
	18–24	1.78 (0.74, 4.28)	
	≥25	1.00 (ref)	
	**Gender** (*n* = 252)		0.045
	Female	0.42 (0.18, 0.97)	
	Male	1.00 (ref)	
	**Geographic place of origin** (*n* = 254)		0.22
	Connecticut	0.39 (0.092, 1.65)	
	Other state	0.31 (0.090, 1.05)	
	Outside US	1.00 (ref)	
	**School** (*n* = 261)		0.24
	Medical	2.92 (0.66, 13.04)	
	Nursing	1.80 (0.31, 10.28)	
	Physician Assistant	1.00 (ref)	
	**Year in Medical School** (*n* = 162)		0.90
	1	2.03 (0.38, 10.95)	
	2	1.15 (0.18, 7.46)	
	3	1.73 (0.26, 11.38)	
	4	1.60 (0.29, 8.93)	
	5 (research year)	1.00 (ref)	

^a^Disagreement is defined as: Strongly disagree, Disagree, or Neither agree nor disagree

Compared with men, women were 0.4 times as likely to disagree with the statement that “it is important to understand this issue [pollution, climate change, and health] because physicians/nurses/physician assistants have an important role to play in educating patients and the public about the impacts of pollution and climate change on health” (i.e., women were more likely to agree with this statement; *p* = 0.0038). Compared with physician assistant students, medical students were 4.55 times as likely to disagree with this statement (i.e., physician assistant students were substantially more likely to agree with this statement; *p* = 0.0008). Finally, compared with men, women were 0.42 times as likely to disagree with the statement that “physicians and health professionals have a responsibility to conserve resources and prevent pollution within their professional practice (i.e., women were more likely to agree with this statement; *p* = 0.045).

### Multivariate logistic regression models

Among the associations that were significant according to the Bonferroni-corrected Fisher’s exact test and the a priori relationships of interest, four involved gender, and in all cases, women exhibited a greater commitment than men to address issues of pollution, climate change, and resource conservation in patient care and professional practice. Because medical students were less likely than nursing and physician assistant students to be female (Table 1), to check whether the gender effect was confounded by professional school, we conducted four multivariate logistic regression analyses that included both gender and school as predictors (one model for each of the four outcomes; Table 8). Although the gender associations were attenuated in the presence of school, two of the associations remained significant (*p* < 0.05) and the magnitude of the point estimate for each association remained meaningful (i.e., 2.35, 0.39, 0.55, 0.45). In addition, we observed an independent effect of school, in which medical students tended to exhibit a lesser commitment than physician assistant students to address issues of pollution, climate change, and resource conservation in patient care and professional practice, with nursing students intermediate.

**Table 8:** Multivariate logistic regression models with gender and school as predictors for each outcome

Multivariate logistic regression models with gender and school as predictor variables			
**Outcome variable**	**Predictor variable**	**Odds ratio (95% CI)**	***p*-value**
**Disagreement**^a^**with statement:*It is important to understand this issue [pollution, climate change, and health], but it isn’t pertinent to patient care.*** (*n* = 252)	**Gender**		0.0064
	Female	2.35 (1.27, 4.34)	
	Male	1.00 (ref)	
	**School**		0.043
	Medical	0.30 (0.12, 0.77)	
	Nursing	0.38 (0.13, 1.11)	
	Physician Assistant	1.00 (ref)	
**Disagreement**^a^**with statement*: It is important to understand this issue [pollution, climate change, and health], so I can help my patients*** (*n* = 252)***.***	**Gender**		0.002
	Female	0.39 (0.21, 0.71)	
	Male	1.00 (ref)	
	**School**		0.20
	Medical	1.72 (0.77, 3.84)	
	Nursing	0.93 (0.34, 2.53)	
	Physician Assistant		
**Disagreement**^a^**with statement:*It is important to understand this issue [pollution, climate change, and health] because physicians/nurses/physician assistants have an important role to play in educating patients and the public about the impacts of pollution and climate change on health.*** (*n* = 252)	**Gender**		0.078
	Female	0.55 (0.28, 1.07)	
	Male	1.00 (ref)	
	**School**		0.018
	Medical	4.96 (1.44, 17.04)	
	Nursing	2.29 (0.55, 9.57)	
	Physician Assistant	1.00 (ref)	
**Disagreement**^a^**with statement:*Physicians and health professionals have a responsibility to conserve resources and prevent pollution within their professional practice.*** (*n* = 252)	**Gender**		0.10
	Female	0.45 (0.18, 1.16)	
	Male	1.00 (ref)	
	**School**		0.62
	Medical	2.14 (0.46, 9.91)	
	Nursing	2.00 (0.34, 11.73)	
	Physician Assistant	1.00 (ref)	

^a^Disagreement is defined as: Strongly disagree, Disagree, or Neither agree nor disagree

### Open-ended comments

At the end of the survey, there was the opportunity for students to provide feedback on the survey or additional comments in a question entitled “optional comments / feedback.” Given that only 19 of the 280 respondents provided comments and that the primary purpose of the survey was to perform a quantitative assessment, we did not conduct in-depth analyses of these comments. However, all responses are shown in the Supplemental Material, and some themes are described here.

Notable themes that arose reflected a desire for institutional level action; existing time pressures in health professionals’ educational curricula; and a newfound understanding of the relationship between climate change, pollution, resource utilization, and healthcare. A medical student stated that “*I think this should be an institutional issue (i.e., the hospital administration) rather than an individual physician issue. I don’t think individuals have nearly the potential for impact that healthcare institutions do.*” One nursing student stated that s/he *“would love for this to be incorporated into our curriculum, but not at the expense of including other equally important education on issues relating to health equity, race, class and gender politics.”* A physician assistant student stated that *“I think this is a very interesting survey that has just started me thinking about healthcare practices in a new light. Nothing regarding healthcare related pollution or environmental effects was covered in our curriculum.”*

## Discussion

While the findings are limited to the context of one university, the results of this study suggest that health professional students are engaged with the concept of environmental stewardship in clinical practice but require improved education and institutional support*.* Most (90%) survey respondents felt that physicians, nurses, and physician assistants have a responsibility to conserve resources and prevent pollution within their professional practice (Table 2), which is similar to the finding from a National Medical Association (NMA) survey of physicians that 78% of respondents “feel that actions they can take in their personal and professional lives can contribute to effective action on climate change” [17]. This suggests that health professional students and physicians feel their actions have the capacity to mitigate the health impacts of pollution and waste generated from the health care industry and that they are concerned about these health impacts. However, although the majority of respondents felt it important to understand the connections between health care sector pollution, climate change, and health, fewer than one-third (29%) of respondents were able to accurately assess the magnitude of health care pollution and its impact on human health (Table 2).

This lack of understanding suggests a need for improved education for health professionals on these issues. Around two-thirds (63%) of respondents to this survey felt that the relationship between pollution, climate change, and health should be covered in the classroom and reinforced in the clinical setting (Table 3). This is similar to the finding from the NMA survey that 80% of the physician respondents felt that teaching about climate change and its associated health impacts should be integrated into medical education [17]. Additionally, around three-quarters (77%) of respondents to our survey felt that health professionals have an important role to play in educating patients and the public about the impacts of pollution and climate change on health (Table 3). This is similar to the finding from the survey of NMA physicians that “a majority of respondents said that physicians have a responsibility to bring the health effects of climate change to the attention of their patients (75%) and the public (71%)” [17].

The similarities in these findings suggest that there is a need to include education on pollution, climate change and health in health professional education, both at Yale and nationally [23]. This education should train health professionals to understand the complex ways in which climate change and pollution impact health [31], how to effectively communicate these health impacts to patients, and how to conserve resources and prevent pollution in clinical practice. Education about the magnitude of disease burden stemming from the health care sector is also needed. Given existing time pressures in health professionals’ education, it will be important to incorporate this education into existing themes in medical education, such as professionalism, quality of care, and safety. In addition, resource conservation practices should be modelled by clinical instructors.

Women were more likely than men, and physician assistant students were more likely than medical students, to believe that 1) the issue of pollution, climate change and health is pertinent to patient care; 2) it is important to understand this issue in order to be able to help patients; 3) it is important to understand this issue because health professionals have an important role to play in educating patients and the public about the impacts of pollution and climate change on health; and 4) health professionals have a responsibility to conserve resources and prevent pollution within their professional practice (Tables 6 and 7). The effects of gender and school appeared to be independent, suggesting that both males and medical students need to be targeted for education about this issue. However, due to the relatively small sample sizes for physician assistant and nursing students, with only one of 53 nursing students and eight of 46 physician assistant students being male, further work is needed to more definitively understand these relationships.

This education should be paired with the implementation of institutional systems that encourage and incentivize resource conservation by staff. Clearly defined policies, procedures, purchasing practices and a motivational culture within hospitals comprise examples of organizational support for resource conservation and pollution prevention efforts [32, 33]. Changing institutional practices to develop systems and places that promote healthful decisions for people and the environment as the default option is one of the most effective ways to motivate behavior change [34]. Institutional level policies that warrant attention include purchasing practices, particularly regarding single-use versus reusable devices; benchmarking clinician utilization and wasteful behaviors around medical device and drug use; and renewable energy sourcing for hospital buildings and operations [35]. Incorporating environmental stewardship into clinical practice as well as institutional systems and decision-making aligns with the concept of *Health in All Policies* that is supported by the American Public Health Association and elucidates the linkages between health, equity, and sustainability [36].

Development and implementation of evidence-based recommendations to minimize unnecessary procedures and services will reduce pollution, improve patient safety, and alleviate some of the time pressures individual health care professionals face. In order to develop these recommendations, research on the pollution and disease burden associated with specific clinical care pathways and medical supplies will be needed. This will require industry transparency about the environmental footprint of various aspects of the health care sector. Tracking medical device and supply utilization will also help guide and improve resource conservation, as doing so will enable individual practitioners and institutions to better understand their comparative supply usage and environmental emissions patterns. Environmental performance should be paired with patient outcome and costs, all as different aspects of quality improvement [35].

Increasing reliance on reusable medical devices when clinically appropriate may also promote resource conservation. There has been a trend towards single-use disposable equipment that may be due to several factors, including hospital managers wishing to prevent a citation from an oversight body such as the Joint Commission [37]. This management trend may be inflating health professionals’ perception of the infection risk associated with reusable devices, as suggested by the 43% of survey respondents who agree or strongly agree that there is strong evidence that single-use disposable medical devices meaningfully reduce infection risk despite a lack of evidence to support this belief. (Interestingly, older students were more likely to disagree.) This is significant given that 53% of respondents ranked infection control as the most important factor in influencing medical device preference.

Although half of survey respondents expressed concern over the infection risk of reusable devices, around half also claimed they prefer reusable devices, with only one-fifth preferring disposable devices. This preference for reusable devices was also found in a survey of US obstetricians and gynecologists on attitudes towards global warming and medical waste, which found that 66% of respondents favored reusable surgical tools over disposable tools when clinically equivalent [20].

Given that infection control is the leading factor influencing medical device preference for survey respondents, education on this issue and institutional commitment to reusable devices when clinically appropriate may be an important way to promote resource conservation. Several studies demonstrate that reusable devices are less environmentally harmful than disposable devices [7, 38, 39]. Research may be warranted to compare the infection risk of reusable versus disposable devices in different settings. However, industry should be incentivized to better design equipment for ease of cleaning so as to reduce the need for disposable devices, given finite planetary resources.

### Study limitations

Given the response rate of only 28%, there was the potential for selection bias, wherein those who had previous awareness of or strong feelings about issues covered by the survey may have been more likely to respond to the survey than those who are unaware of or feel indifferent towards these issues. The survey was exclusively distributed to student email inboxes, and it is possible that students with stronger opinions may have been more likely to pay attention to requests to complete the survey. The response rate of 28% is similar to the 30% response rate for the NMA survey of physicians [17]; higher than the 17% response rate for an American Thoracic Society Member Survey [18] and the 22% response rate for an American Academy of Allergy Asthma and Immunology survey [22]; and lower than the 42% response rate for an American Society of Anesthesiologists survey [21], the 70% response rate for a University of Pittsburgh Medical Center Magee Women’s Hospital obstetricians and gynecologists survey [20], and the 96.6% response rate for the Chinese medical student survey [25]. Given the large number of emails that medical, nursing, and physician assistant students regularly receive, it is possible that a higher response rate could have been achieved had additional methods of recruitment been employed.

The survey questions were pilot tested with medical students, but not with nursing or physician assistant students, which may have contributed to some confusion in question interpretation. Some respondents utilized the open-ended comments to express confusion regarding wording of some of the questions. Future surveys should modify the language of certain questions to eliminate conjunctions to improve respondent clarity.

The small number of optional open-ended responses prevented an in-depth analysis of the comments. Future research could focus on developing stronger qualitative questions regarding health professional students’ understanding of the relationships between climate change, pollution, and resource utilization in health care.

For the most part, the low sample sizes for the nursing and physician assistant students prevented discernment of statistically significant response differences among medical, nursing, and physician assistant students. Future research could aim for larger sample sizes that would facilitate identification of differences that may exist among these groups.

This study did not assess race and ethnicity. Future studies could examine possible relationships between race and ethnicity and opinions on climate, pollution and resource conservation.

Because our study was confined to one institution, our results may not be representative of the broader US population of health professional students; future research could expand upon this study to determine whether these findings are generalizable to other institutions.

## Conclusions

This study contributes to the literature on health professional students’ understanding of the relationships between climate change, pollution, and resource conservation in health care. The findings suggest that medical, nursing, and physician assistant students are engaged with the concept of resource conservation and pollution prevention in clinical practice but require improved education and institutional support in order to effectively act as environmental stewards. Most are concerned about the health impacts of climate change and about health care pollution, feel that understanding this issue is important for patient care, and believe it is their duty to conserve resources and prevent pollution within their professional practice. Incorporating education on how practices within the health care sector contribute to pollution, as well as about the global issue of climate change and human health, into medical, nursing, and physician assistant student training will help raise awareness for professional responsibility and increase preparedness. Education on what can be done to reduce pollution and greenhouse gas emissions and promote resource conservation within the health care sector at the individual and systems levels is also necessary. For this education to be effective, increased industry transparency on the environmental footprint of the health care sector and research regarding the environmental footprint of specific clinical practices and supply chains will be required.

## Data Availability

The datasets used and/or analyzed during the current study are available from the corresponding author on request by email (emma_c.ryan@tufts.edu).

## Abbreviation

NMA: National Medical Association

## References

[1] Landrigan PJ, Fuller R, Acosta NJR, Adeyi O, Arnold R, Basu N, Baldé AB, Bertollini R, Bose-O'Reilly S, Boufford JI (2017). The lancet commission on pollution and health. Lancet.

[2] Costello A, Abbas M, Allen A, Ball S, Bell S, Bellamy R, Friel S, Groce N, Johnson A, Kett M (2009). Managing the health effects of climate change. Lancet.

[3] Watts N, Adger WN, Agnolucci P, Blackstock J, Byass P, Cai W, Chaytor S, Colbourn T, Collins M, Cooper A (2015). Health and climate change: policy responses to protect public health. Lancet.

[4] World Health Organization (2018). COP24 special report: health and climate change.

[5] Intergovernmental Panel on Climate Change. Global Warming of 1.5°C: An IPCC special report on the impacts of global warming of 1.5°C above pre-industrial levels and related global greenhouse gas emission pathways, in the context of strengthening the global response to the threat of climate change, sustainable development, and efforts to eradicate poverty. Summary for policymakers. Switzerland; 2018.

[6] Eckelman MJ, Sherman JD (2017). Estimated global disease burden from US health care sector greenhouse gas emissions. Res Pract.

[7] Sherman JD, Raibley LA, Eckelman MJ (2018). Life cycle assessment and costing methods for device procurement: comparing reusable and single-use disposable laryngoscopes. Anesth Analg.

[8] Sherman JD, Hopf HW (2018). Balancing infection control and environmental protection as a matter of patient safety: the case of laryngoscope handles. Anesth Analg.

[9] Kohn LT, Corrigan J, Donaldson MS (2000). To err is human: building a safer health system.

[10] Schoen J, Chopra V (2018). The harm we do: the environmental impact of medicine. J Hosp Med.

[11] Frumkin H. The US health care sector’s carbon footprint: stomping or treading lightly? Am J Public Health. 2018;108(52;Suppl2):S56-7. 10.2105/AJPH.2017.304160 PMC5922200 29072935

[12] Sarfaty M, Abouzaid S (2009). The physician's response to climate change. Fam Med.

[13] Macpherson CC, Hill J (2017). Are physicians obliged to lead environmental sustainability efforts in health care organizations?. AMA J Ethics.

[14] Leiserowitz A, Maibach E, Roser-Renouf C, Rosenthal S, Cutler M, Kotcher J. Climate change in the American mind: October 2017. New Haven, CT: Yale Program on Climate Change Communication: Yale University and George Mason University; 2017.

[15] Maibach EW, Kreslake JM, Roser-Renouf C, Rosenthal S, Feinberg G, Leiserowitz AA (2015). Do Americans understand that global warming is harmful to human health? Evidence from a national survey. Ann Glob Health.

[16] The Medical Society Consortium on Climate and Health. In: https://medsocietiesforclimatehealthorg. Accessed 20 Apr 2019.

[17] Sarfaty M, Mitchell M, Bloodhart B, Maibach EW. Key findings of a National Medical Association physician survey. National Medical Association, George Mason University Center for Climate Change Communication; 2014.

[18] Sarfaty M, Bloodhart B, Ewart G, Thurston GD, Balmes JR, Guidotti TL, Maibach EW (2015). American Thoracic Society member survey on climate change and health. Am Thorac Soc Rep.

[19] Sarfaty M, Mitchell M, Bloodhart B, Maibach EW (2014). A survey of African American physicians on the health effects of climate change. Int J Environ Res Public Health.

[20] Thiel C, Duncan P, Woods N (2017). Attitude of US obstetricians and gynaecologists to global warming and medical waste. J Health Serv Res Policy.

[21] Ard JL, Tobin K, Huncke T, Kline R, Ryan SM, Bell C (2016). A survey of the American Society of Anesthesiologists regarding environmental attitudes, knowledge, and organization. A & A Case Rep..

[22] Sarfaty M, Kreslake JM, Casale TB, Maibach EW (2016). Views of AAAAI members on climate change and health. J Allergy Clin Immunol Pract.

[23] Teherani A, Nishimura H, Apatira L, Newman T, Ryan S (2017). Identification of core objectives for teaching sustainable healthcare education. Med Educ Online.

[24] Hamel Green EI, Blashki G, Berry HL (2009). Preparing Australian medical students for climate change. Aust Fam Physician.

[25] Liao W, Yang L, Zhong S, Hess JJ, Wang Q, Bao J, Huang C (2018). Preparing the next generation of health professionals to tackle climate change: are China's medical students ready?. Environ Res.

[26] Wellbury C, Sarfaty M, Kreslake J. A stepped approach towards developing a multi-institutional climate change and health curriculum. Presentation to AMSA; 2016.

[27] Maxwell J, Blashki G. Teaching about climate change in medical education: an opportunity. J Public Health Res. 2016;5(1):673. 10.4081/jphr.2016.673 PMC4856872 27190980

[28] Bell EJ (2010). Climate change: what competencies and which medical education and training approaches?. BMC Med Educ.

[29] Wellbery C, Sheffield P, Timmireddy K, Sarfaty M, Teherani A, Fallar R (2018). It's time for medical schools to introduce climate change into their curricula. Acad Med.

[30] Yale University Office of Institutional Research Historical Data. In*.* https://oir.yale.edu/historical-data-main-page: Yale University: Accessed April 20, 2019. 2018.

[31] Yang L, Liao W, Liu C, Na Z, Shuang Z, Huang C (2018). Associations between knowledge of the causes and perceived impacts of climate change: a cross-sectional survey of medical, public health and nursing students in universities in China. Int J Environ Res Public Health.

[32] Kallio H, Pietilä A-M, Johnson M, Kangasniemi M (2018). Environmental responsibility in hospital care: findings from a qualitative study. J Hosp Admin.

[33] Singleton JA, Lau ETL, Nissen L. The impact of drugs on the environment: what do pharmacists and technicians believe? *8th FIP World Congress of Pharmacy and Pharmaceutical Sciences.* Glasgow: (Unpublished); 2018.

[34] Farley T, Cohen DA (2005). Prescription for a healthy nation: a new approach to improving our lives by fixing our everyday world.

[35] Sherman JD, MacNeill A, Thiel C (2019). Reducing pollution from the health care industry. JAMA.

[36] Rudolph L, Caplan J, Ben-Moshe K, Dillon L (2013). Health in all policies: a guide for state and local governments.

[37] Morey TE, Sappenfield JW, Gravenstein N, Rice MJ (2015). Joint Commission and regulatory fatigue/weakness/overabundance/distraction: clinical context matters. Anesth Analg.

[38] Eckelman M, Mosher M, Gonzalez A, Sherman J (2012). Comparative life cycle assessment of disposable and reusable laryngeal mask airways. Anesth Analg.

[39] Sanchez SA, Eckelman MJ, Sherman JD (2020). Environmental and economic comparison of reusable and disposable blood pressure cuffs in multiple clinical settings. Resour Conserv Recycling.

